# Nurturing Minds: How Maternal Investment Impacts Cognitive Aging and Dementia Risk in Men and Women

**DOI:** 10.1002/alz70860_106754

**Published:** 2025-12-23

**Authors:** Kimson Johnson, Maddison Linker

**Affiliations:** ^1^ University of Michigan Institute for Social Research, Ann Arbor, MI, USA; ^2^ University of Michigan, Ann Arbor, MI, USA

## Abstract

**Background:**

Early‐life environments shape brain development and cognitive function, with lasting effects into adulthood. While research highlights early‐life influences on late‐life health, the specific impact of maternal involvement and investment in a child's upbringing on dementia risk remains unclear. Additionally, how these influences differ between older men and women is understudied.

A nurturing and engaged maternal presence, characterized by time, effort, and attention to a child's development before age 18, plays a crucial role in shaping cognitive health. Mothers who actively teach, provide guidance, and invest in their child's learning may contribute to stronger cognitive outcomes throughout life. This study examines how maternal involvement and investment in childhood influence cognitive functioning in later life and explores potential gender differences in these associations.

**Method:**

Latent Class Analysis (LCA) was used to identify distinct patterns of maternal investment (maternal time, attention, and effort) using data from the Health and Retirement Study (HRS), including the Childhood Health and Family, Life History, and 2020 Core datasets (*n* = 7,656; Women: 4,720 and Men: 2,936) of adults aged 51+. Cognitive functioning was assessed using the 2020 total word recall summary score (range: 0‐20; *M*women:10.25 (SD:3.76), *M*men:9.22 (SD:3.61)). The analysis accounted for variables related to age, household size, and childhood health status. Gender‐stratified analyses were conducted to explore differences in the associations between early‐life family environments and dementia risk.

**Result:**

Among maternal investment classes, four distinct groups were identified (Intensive, Engaged, Minimal, and No Investment). Intensive Maternal Investment (*n* = 4,826; Women: 2,871, Men: 1,955) was the largest group. Compared to other groups, intensive maternal investment was significantly associated with higher cognitive functioning (β=0.20, *p* < .05), particularly for men (β=0.20, *p* < .05).

**Conclusion:**

This study highlights the importance of maternal investment in early‐life cognitive development, with implications for interventions that enhance cognitive health in later life. Tailored approaches addressing gender‐related early‐life differences may improve late life outcomes for both men and women.

